# Efficacy and safety of extracorporeal shockwave therapy in chronic low back pain: a systematic review and meta-analysis of 632 patients

**DOI:** 10.1186/s13018-023-03943-x

**Published:** 2023-06-24

**Authors:** Kun Liu, Qingyu Zhang, Lili Chen, Haoran Zhang, Xiqiang Xu, Zenong Yuan, Jun Dong

**Affiliations:** 1 Shandong Sport University, No.10600, Road Century, Jinan, 250102 Shandong China; 2 Department of Orthopedics, Shandong Provincial Hospital Affiliated to Shandong First Medical University, No.324, Road Jing Wu Wei Qi, Jinan, 250021 Shandong China

**Keywords:** Chronic low back pain, Dysfunction, ESWT, Mental health, Meta-analysis

## Abstract

**Background:**

Extracorporeal shock wave therapy (ESWT) has been widely used for pain control in musculoskeletal disorders. Whether ESWT can relieve chronic low back pain (CLBP) and improve lumbar function is still unclear. Therefore, we conducted a meta-analysis of relevant studies to comprehensively analyse and determine the efficacy and safety of ESWT for chronic low back pain.

**Methods:**

Four databases were systematically searched for randomized controlled trials (RCTs) on ESWT for CLBP. The quality of the included studies was evaluated according to Cochrane systematic review criteria, relevant data were extracted, and meta-analysis was performed using RevMan 5.4 software. The primary outcomes were pain intensity, disability status, and mental health. The data were expressed as standardized mean differences (SMD) or weighted mean difference (WMD) and 95% confidence intervals (CI). Heterogeneity was assessed using the *I*^2^ statistic. If *I*^2^ ≥ 50%, a random effects model was applied; otherwise, a fixed effects model was used.

**Results:**

Twelve RCTs involving 632 patients were included in this meta-analysis. The ESWT group reported significantly more pain relief than the control group at 4 weeks (WMD =  − 1.04; 95% CI =  − 1.44 to − 0.65; *P* < 0.001) and 12 weeks (WMD =  − 0.85; 95% CI =  − 1.30 to − 0.41; *P* < 0.001). Regarding the dysfunction index, ESWT led to significant improvement in lumbar dysfunction compared with the control group at 4 weeks (WMD =  − 4.22; 95% CI =  − 7.55 to − 0.89; *P* < 0.001) and 12 weeks (WMD =  − 4.51; 95% CI =  − 8.58 to − 0.44; *P* = 0.03). For mental health, there was no significant difference between the ESWT group and the control group after 4 weeks of intervention (SMD = 1.17; 95% CI =  − 0.10 to 2.45; *P* = 0.07).

**Conclusion:**

This systematic review and meta-analysis found that ESWT provided better pain relief and improved lumbar dysfunction compared with the other interventions included, and no serious adverse effects were found. There was no significant effect of ESWT on the mental health of patients, but we hope to obtain more RCTs for further analysis in the future. Based on the pooled results, we suggest that ESWT is effective and safe for treating chronic low back pain.

**Supplementary Information:**

The online version contains supplementary material available at 10.1186/s13018-023-03943-x.

## Introduction

CLBP refers to pain that lasts for at least 12 weeks and occurs in the area below the margin of the low ribs, above the transverse hip line and between the bilateral midaxillary line; this pain is usually accompanied by pain symptoms in one or both lower limbs [1]. The global prevalence rate of CLBP is 13.1~20.3%, and it has been increasing in the past decade, with the number of affected patients rising from 370 million in 1990 to 570 million in 2017 [2]. CLBP has become a global public health problem due to its high incidence, long course and easy recurrence, which seriously affects the quality of life of patients and even causes adverse psychological effects [3]. Pain and limitation of movement are its most basic symptoms. Pain alters the contraction pattern of the trunk muscles, resulting in spasm, increased tone and even atrophy of the low back muscles, thereby significantly reducing the ability of the muscles to engage and destabilize the spine and vertebral balance [4, 5]. In addition, prolonged poor posture in the low back can lead to fatigue of the low back muscles and oedema of the surrounding soft tissues, exudation of inflammatory cells, accumulation of metabolic products and degeneration of muscle fibres, resulting in local adhesions, chronic hypoxia of the muscles and pain; all of these symptoms can contribute to recurrent episodes of CLBP [6, 7].

At present, CLBP is mainly treated conservatively (e.g. physical exercise, physiotherapy, drugs and other nonsurgical therapy) with the purposes of relieving pain and restoring physical function [8–10]. However, as a self-exercise therapy, physical exercise has shortcomings such as lack of standard posture and poor adherence; physiotherapy has difficulty achieving a long-term analgesic effect, while drug treatment may be accompanied by nausea, constipation, fatigue and other side effects [11, 12]. Most guidelines advocate the use of non-steroidal anti-inflammatory drugs (NSAIDs) in CLBP, but their long-term efficacy is unknown and the effectiveness of NSAIDs may be overestimated [13, 14]. Recently, mesenchymal stem cells appear to have shown good results in relieving degenerative discogenic pain, but its scope of application is still limited and safety needs further confirmation [15]. In addition, despite the availability of various interventions, more than two-thirds of patients with low back pain relapse within 12 months of recovery [16]. Therefore, it is particularly important to seek other safe and effective treatment strategies.

As an emerging therapeutic method, ESWT is a series of single sound pulses characterized by a high pressure peak and short-term rapid pressure rises and has achieved significant results in the treatment of musculoskeletal system diseases such as osteonecrosis of the femoral head and myofascitis [17, 18]. However, the use of ESWT in the treatment of CLBP is still controversial, and some clinical guidelines do not recommend it as a routine choice [19]. In recent years, some RCTs have focused on the use of ESWT in the treatment of CLBP. Therefore, an updated meta-analysis is needed to synthesize the literature. The main purpose of this meta-analysis was to evaluate the efficacy and safety of ESWT in reducing pain, improving lumbar function, and promoting mental health in patients with CLBP compared with other treatment methods, such as physical exercise, physiotherapy, and drugs.

## Methods

### Design

This systematic review and meta-analysis was based on the Cochrane Handbook of Systematic Reviews on Interventions [20] and strictly followed the recommended reporting items for the Preferred Reporting Items for Systematic Reviews and Meta-Analyses (PRISMA) statement guidelines [21]. The review protocol was registered in the PROSPERO database (registration number: CRD42023421589).

### Search strategy

The PubMed, Embase, Web of Science, and Cochrane Library databases were systematically searched from the initial release of the relevant database until April 25, 2023, to identify studies related to the use of ESWT for CLBP. The following search terms were used in the initial literature search: (Extracorporeal Shock Wave Therapy or ESWT) and (Chronic Low Back Pain or low back pain). Two researchers (KL and LLC) independently reviewed the selected studies, and any disagreements were resolved through discussion with a third senior investigator (DJ). In addition, the reference lists of these articles were manually checked to identify other publications that might be relevant.

### Inclusion criteria

The inclusion criteria were as follows: (1) Adult patients (aged ≥ 18 years): clinically diagnosed with CLBP of mechanical origin; (2) Experimental group: ESWT or ESWT combined with other intervention methods; (3) Control group: physical exercise, physiotherapy, medicine or other similar interventions; (4) Results: Visual analogue scale (VAS), Oswestry Disability Index (ODI), mental health and other functional parameters; (5) Study design: Randomized controlled trial (RCT).

### Exclusion criteria

The exclusion criteria were as follows: (1) nonhuman research or animal experiments; (2) studies that included participants with post-spinal surgery, pregnancy, or other spinal conditions (fractures, tumours, spondylolisthesis, ankylosing spondylitis, severe osteoporosis, cauda equina syndrome); (3) articles such as abstracts, letters, editorials, expert opinions, comments, and case reports; (4) non-English studies; and (5) studies without suitable data for analysis.

### Data extraction

The demographic characteristics extracted included first author, year of publication, study design, sample size of each study, mean age of patients, sex ratio, and follow-up. The main outcome measures of the treatment effect in this study included the VAS pain score, ODI dysfunction index and patients' mental health score. The main mental health scales include the 36-Item Short Form Health Survey (SF-36), Patient Health Questionnaire 9 (PHQ-9) and Beck Depression Index (BDI). If scores were recorded at different follow-up times, we chose the time points closest to 4 weeks and 12 weeks to predict efficacy. In addition, adverse events were recorded.

### Bias assessment and quality classification

The quality of the included studies was assessed by the version 2 of the Risk of Bias tool of the Cochrane Library (RoB 2) [22]. Seven domains of bias, including selection bias, performance bias, detection bias, attribution bias, reporting bias, and other sources of bias, were evaluated. Judgements were presented as "high risk," "low risk," or "risk ambiguous," and the quality assessment numbers were generated by RevMan version 5.4. Two independent reviewers (QYZ and XQX) assessed the risk of bias, and a third senior investigator (DJ) resolved cases of disagreement between the former. The GRADE (Grading of Recommendations, Assessment, Development, and Evaluation) method was used to evaluate the overall quality of the evidence based on risk of bias, indirectness, inconsistency, publication bias, imprecision, and other factors. The GRADE method, depending on estimated effects, classifies the quality of evidence as high, moderate, low, or very low [23].

### Statistical analyses

Meta-analysis was performed using RevMan5.4 software provided by the Cochrane Collaboration network, and forest maps were used to display the results. Since the measured data were continuous variables, the SMD or WMD and 95% CI were selected as the main effect parameters according to the differences in the measurement methods of the indicators. Heterogeneity was tested by the P value of *Chi*^2^ and *I*^2^. When statistical heterogeneity was significant (*P* < 0.10 or *I*^2^ > 50%), the random effects model was chosen. When statistical heterogeneity was not significant (*P* ≥ 0.10 or *I*^2^ ≤ 50%), the fixed effects model was adopted. Furthermore, the source of heterogeneity can be explored by sensitivity analysis and subgroup analysis. Subgroup analyses were performed according to follow-up time and intervention method. According to Egger et al. [24] and with more than ten included studies, we assessed the publication bias between the included studies by visual inspection of the funnel plot.

## Results

### Selection of studies

**Fig. 1:**
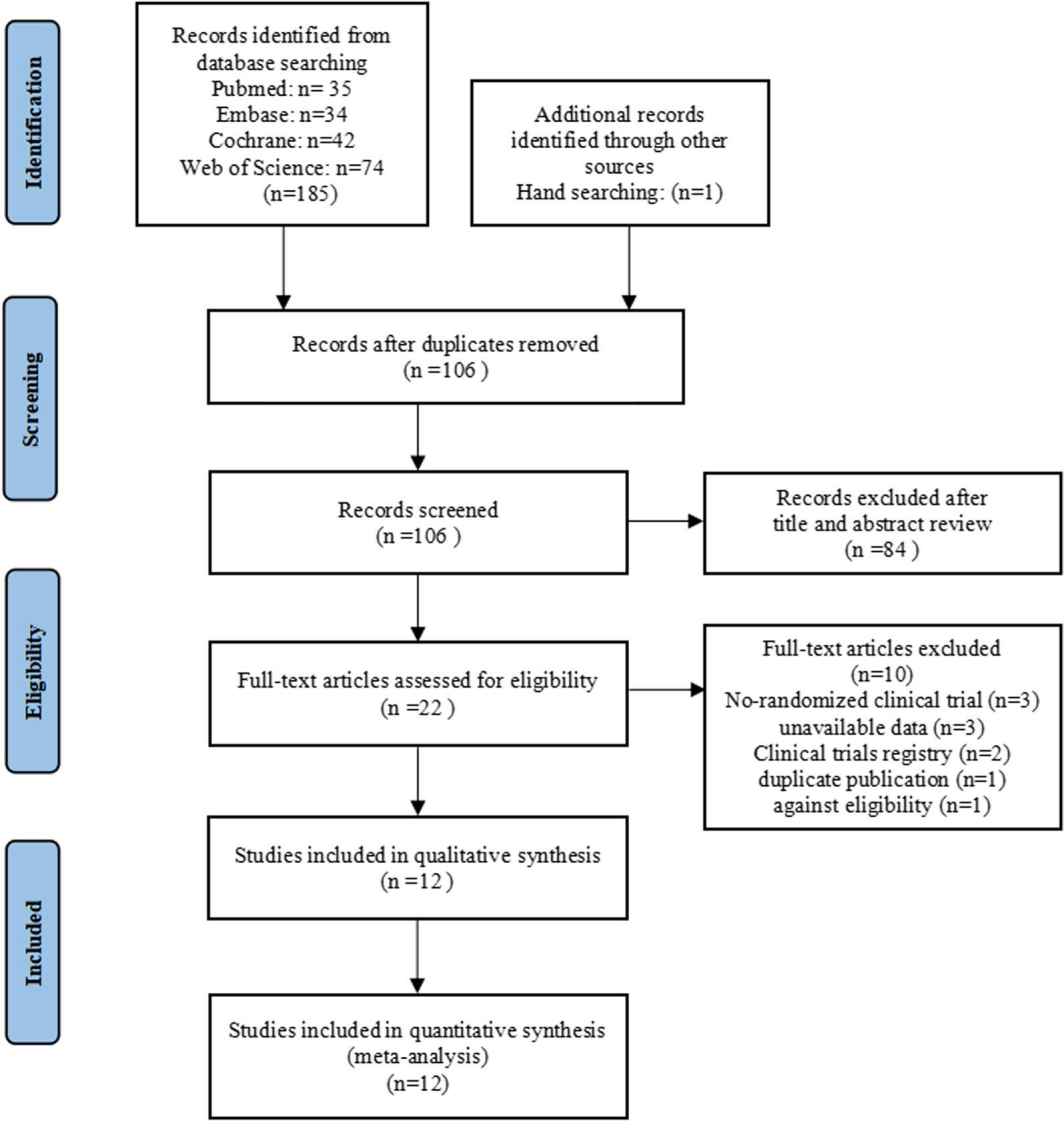
The selection process of this meta-analysis

In the initial literature search, 186 papers were retrieved. We detected and removed 80 duplicate articles using Endnote X9 software. Additionally, 84 studies were excluded after reviewing the titles and abstracts. Then, after a full text review, we excluded ten articles that did not meet the inclusion criteria. Finally, 12 RCTs involving a total of 632 patients (318 in the ESWT group and 314 in the control group) were included in this study. The selection process is presented in the PRISMA flowchart (Fig. 1).

### Study characteristics and risk of bias

**Fig. 2:**
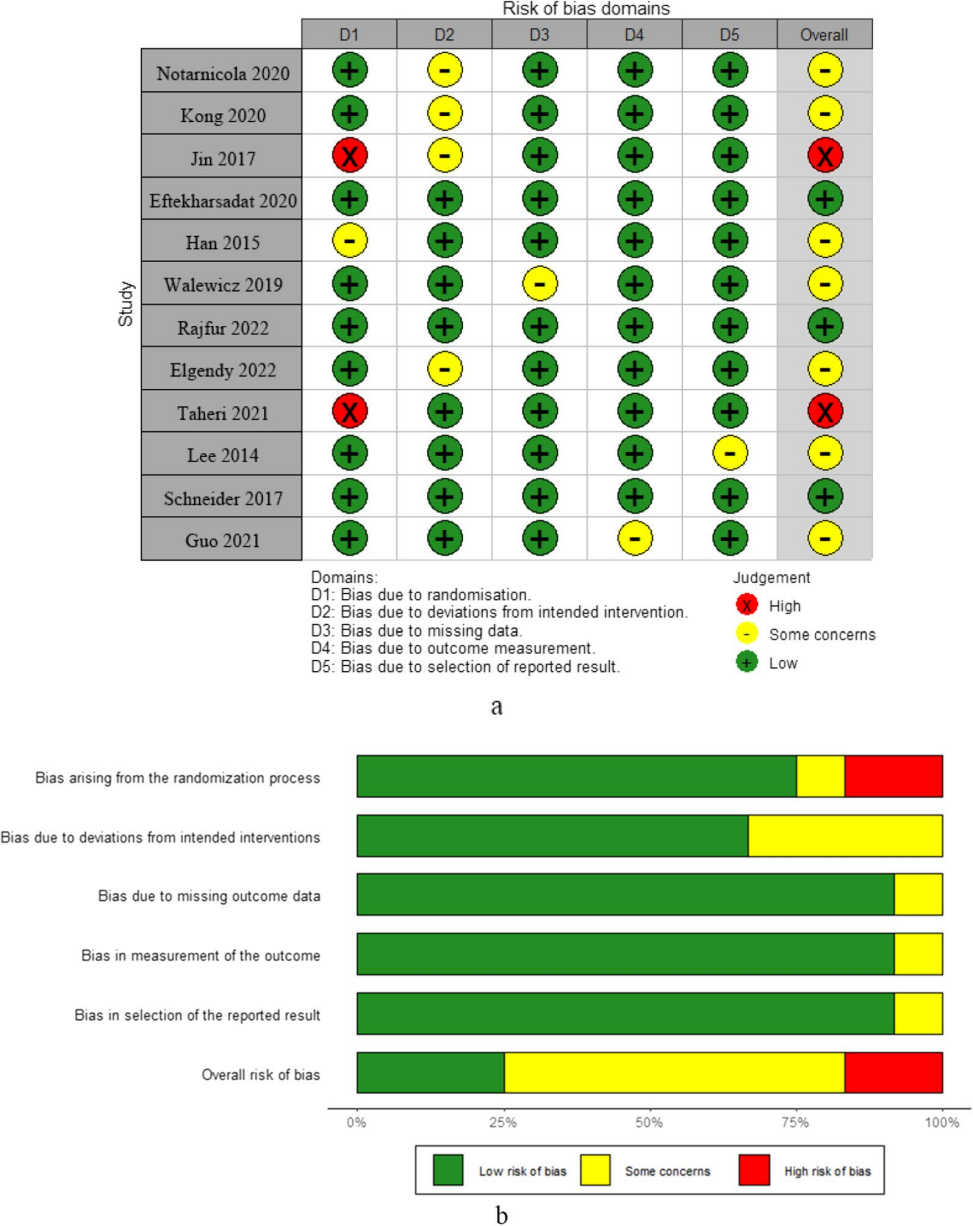
**a** Judgement plots for risk of bias items for each RCT. **b** Weighted summary plot of overall bias type in RCTs

These studies are characterized in Table 1. All articles were published in English between 2014 and 2022. Sample sizes range from 28 to 200. All the experimental groups received ESWT treatment, while the control group received different conservative treatments, including exercise therapy [25–28], physiotherapy [29–31], drug injection [32, 33], oral medication [34, 35], and manual therapy [36]. The items for risk of bias included in each study are shown in Fig. 2. The quality level assessment of the relevant studies is shown in Additional file 1: Table S2.

**Table 1:** Characteristics of the included RCTs

References		Study design	Sample size		Gender (male)		Age (mean ± SD)		Intervention	Follow-up
			ESWT	Control	ESWT	Control	ESWT	Control		
Notarnicola et al. [26]		RCT	15	15	–	–	62.6 ± 11.8	(range: 43.0–82.0)	ESWT: Shockwaves of 2000 pulses in total were applied at 0.03 mj/mm^2^ and a frequency of 4 Hz/s, once per week for three sessions Control: Bridging exercise, adductor ball squeeze exercise, abdominal marching exercise and reverse curl-ups exercise (50 min), twice a week for 4 weeks	12 weeks
Kong et al. [31]		RCT	100	100	60	60	44.45 ± 8.27	44.53 ± 8.19	ESWT: The impulse voltage is set as 7.0–9.0 kv, the impulse energy is 0.1–0.2 mj/mm^2^, and the times of shocks are set as 1200. ESWT was conducted usually for 1–2 courses, 5 times as 1 course Control: Laser therapeutic, once a week, with a total of 4 times	1 year
Jin et al. (2017)		RCT	15	15	8	9	55.46 ± 15.09	53.13 ± 19.62	ESWT: With 2000 shock waves applied at each session at an intensity of 0.085–0.148 mj/mm^2^. We repeated this procedure a total of three times, at 3-day intervals Control: Trigger point injection	4 weeks
Eftekharsadat et al. [33]		RCT	27	27	20	17	44.74 ± 9.34	45.04 ± 11.86	ESWT: Shockwaves of 1500 pulses with an energy flux density of 0.1 mj/mm^2^/min, energy level of 2–4, a frequency of 10–16 Hz, and pulse rate of 160/min in total, once a week for 5 weeks. Physical exercise: stretching exercises Control: Trigger point injection, muscle injection of 40 mg triamcinolone + 2 mL lidocaine 2%, one session. Physical exercise: stretching exercises	4 weeks
Han et al. [30]		RCT	15	15	9		49.7 ± 8.3	46.0 ± 8.9	ESWT: Each patient assumed a prone position, and 1000 shock waves (7 times per sec) were applied at 2.5 Hz at low energy flux densities of 0.01–0.16 mj/mm^2^ using a 17 mm head. The treatment was conducted at the quadratus lumborum muscle and the sacroiliac joint, where the patients complained of pain Control: Bridging exercise, adductor ball squeeze exercise, abdominal marching exercise and reverse curl-ups exercise (50 min), twice a week for 4 weeks	6 weeks
Walewicz et al. [25]		RCT	20	20	6	5	51.1 ± 8.4	55.8 ± 9.3	ESWT: Shockwaves of 2000 pulses with the pressure of 2.5 bars (corresponding to an energy flux density of 0.1 mj/mm^2^), 5 Hz frequency, and treatment time of seven minutes; Physical exercise Control: Sham shockwaves therapy; Physical exercise	12 weeks
Rajfur et al. [27]		RCT	19	18	10	10	43.0 ± 13.1	45.4 ± 14.0	ESWT: Shockwaves of 1000 pulses with an energy flux density of 0.15 mj/mm^2^/min, frequency 4 Hz, twice a week for 5 weeks. Physical exercise Control: Sham shockwaves therapy. Physical exercise	12 weeks
Elgendy et al. [28]		RCT	15	15	10	10	32.73 ± 6.73	33.26 ± 5.48	ESWT: 2000 shocks, 0.10 mj/mm^2^ energy, 5 Hz frequency, using a 17 mm head were administered, twice a week for 6 weeks. Stretching exercises were performed for the hamstrings, iliopsoas, and back extensors Control: Stretching exercises were performed for the hamstrings, iliopsoas, and back extensors	6 weeks
Taheri et al. [34]		RCT	17	15	6	9	42.5 ± 10.1	37.1 ± 11.8	ESWT: Shockwaves of 1500 pulses in total were applied at 0.15 mj/mm^2^ and a frequency of 4 Hz/s, once a week for 4 weeks. Oral medications; stretching exercises Control: Sham shockwaves therapy; Oral medications; stretching exercises	12 weeks
Lee et al. [29]		RCT	13	15	9		53.92 ± 10.38	54.33 ± 13.16	ESWT: Shockwaves of 2000 pulses in total were applied at 0.10 mj/mm^2^ and a frequency of 5 Hz/s, twice a week for 6 weeks; Williams’ exercises and McKenzie’s exercise Control: Hot packs, and ultrasound and electrotherapy; Williams’ exercises and McKenzie’s exercise	6 weeks
Schneider [36]		RCT	15	15	–	–		43.2 (range: 23–65)	ESWT: Impulse parameters: 15–42 Hz; Myofascial trigger therapy, twice a week for 3 weeks Control: Myofascial trigger therapy, twice a week for 3 weeks	12 weeks
Guo et al. [35]		RCT	47	44	22	25	34.9 ± 8.7	36.0 ± 11.2	ESWT: 4000 pulses, 15 Hz, once a week for 4 weeks Control: Medication: celecoxib and eperisone	12 weeks

*ESWT* extracorporeal shock wave therapy, *SD* standard deviation

### Pain score at 4 weeks

**Fig. 3:**
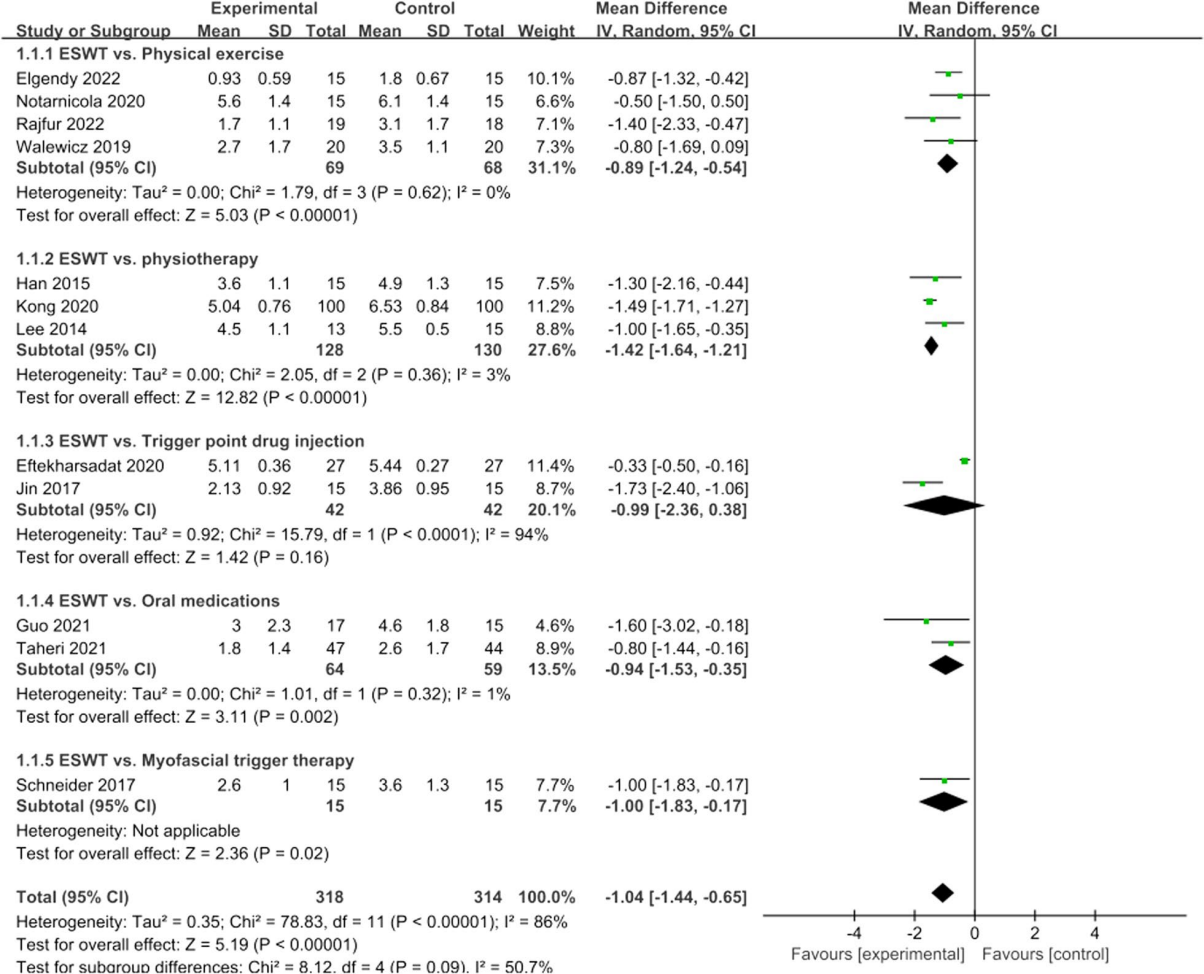
Forest plot of pain score at 4 weeks

Twelve articles included in our study compared pain scores at 4 weeks between the ESWT group and the control group. There was significant heterogeneity (*I*^2^ = 86%, *P* < 0.001), so we conducted subgroup analysis according to the intervention methods of the control group using the random effects model. The results showed that the trigger drug injection group had high heterogeneity (*I*^2^ = 94%, *P* < 0.001), but there was no significant difference between this group and ESWT, so the overall results were still consistent. The present meta-analysis demonstrated that ESWT was associated with a significant reduction in pain score at 4 weeks (WMD =  − 1.04; 95% CI =  − 1.44 to − 0.65; *P* < 0.00001, Fig. 3).

### Pain score at 12 weeks

**Fig. 4:**
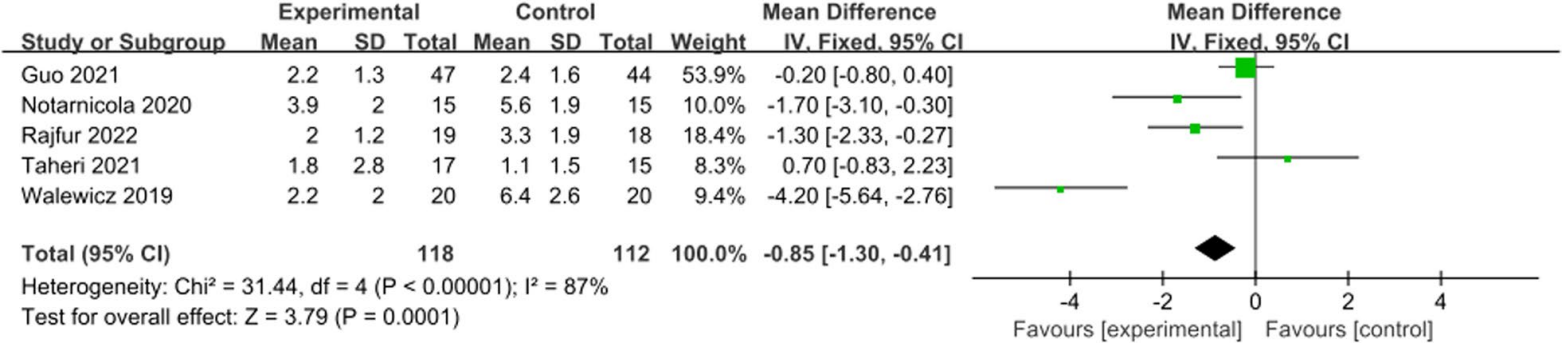
Forest plot of pain score at 12 weeks

A total of five studies reported pain scores at 12 weeks. There was significant heterogeneity, and a random effects model was used (*I*^2^ = 87%, *P* < 0.001). This meta-analysis showed that the pain score of the ESWT group was significantly lower than that of the control group at 12 weeks (WMD =  − 0.85; 95% CI =  − 1.30 to − 0.41; *P* = 0.0001, Fig. 4).

### ODI score at 4 weeks

**Fig. 5:**
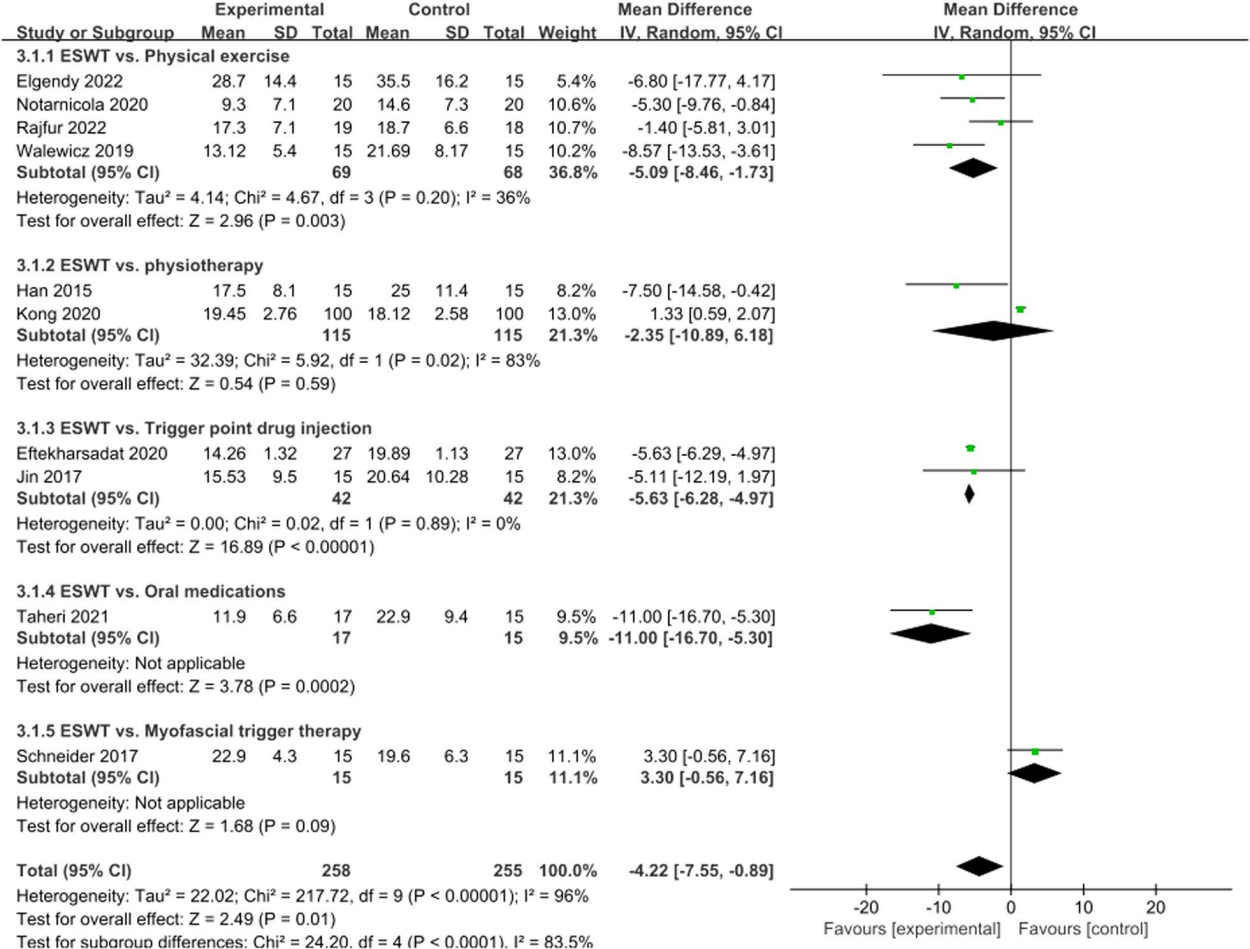
Forest plot of ODI score at 4 weeks

A total of ten articles compared ODI scores at 4 weeks between the ESWT and control groups. The difference was significant (*I*^2^ = 96%, *P* = 0.01). Subgroup analysis was conducted according to the intervention methods of the control group, and a random effects model was selected. The physiotherapy group was the main source of heterogeneity (*I*^2^ = 83%, *P* = 0.02), but there was no significant difference between the control group and the ESWT group, so the overall results were consistent and reliable. In the present meta-analysis, ESWT was associated with a significant increase in ODI scores at 4 weeks (WMD =  − 4.22; 95% CI =  − 7.55 to − 0.89; *P* = 0.01, Fig. 5).

### ODI score at 12 weeks

**Fig. 6:**
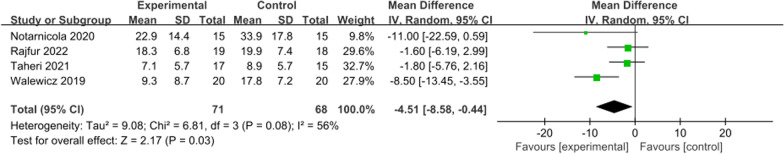
Forest plot of ODI score at 12 weeks

ODI scores at 12 weeks were obtained from four studies with significant heterogeneity (*I*^2^ = 56%, *P* = 0.08), and a random effects model was used. The combined results showed a significant difference between the groups (WMD =  − 4.51; 95% CI =  − 8.58 to − 0.44; *P* = 0.03, Fig. 6).

### Mental health score at 4 weeks

**Fig. 7:**
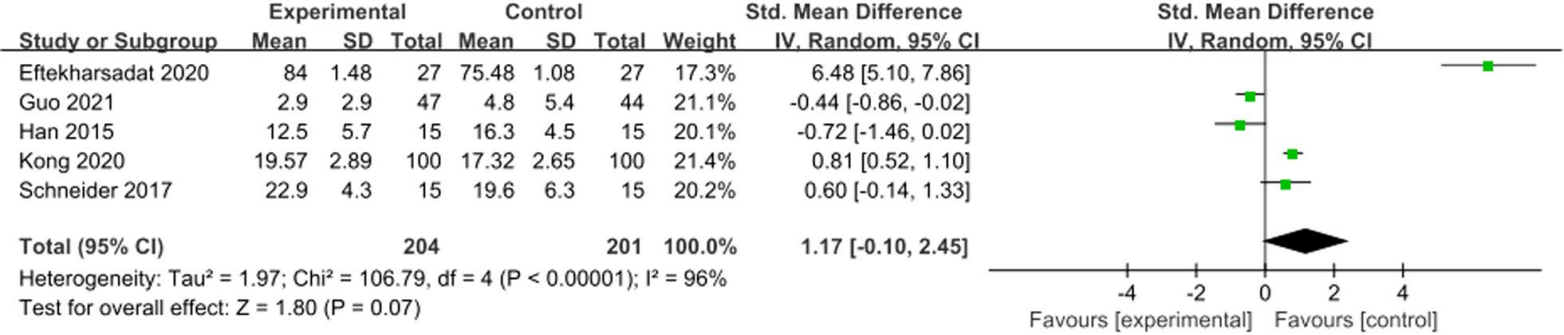
Forest plot of mental health score at 4 weeks

A total of five studies reported mental health scores at 4 weeks, with significant heterogeneity (*I*^2^ = 96%, *P* < 0.001). The questionnaires used for mental health scores were inconsistent, so SMD was selected for meta-analysis. The results showed no specific significant difference in mental health score between the control group and ESWT group at 4 weeks (SMD = 1.17; 95% CI =  − 0.10 to 2.45; *P* = 0.07, Fig. 7).

### Adverse events

No serious adverse reactions were reported in any of the 12 studies; seven studies specifically reported that adverse reactions did not occur, and five studies did not record adverse reactions at all.

### Qualitative analysis

The studies included in this meta-analysis involved both radial extracorporeal shockwave (r-ESWT) and focused extracorporeal shockwave (f-ESWT), so it is of interest to analyse which type of ESWT is more effective for CLBP. Based on the data available in this study, although it is not possible to directly quantify which device and type had a superiority in the treatment, we have obtained some valuable information from other relevant studies. A study of non-calcific rotator cuff tendinopathies showed that f-ESWT was significantly more effective than r-ESWT at long-term follow-up of more than 24 weeks [37]. Another study on knee osteoarthritis also showed the same results [38]. However, DeLuca et al. [39] reported that no significant difference was found between f-ESWT and r-ESWT in terms of efficacy in plantar fasciitis and that most patients could achieve functional gains with either form of shockwave. Which type of ESWT is more advantageous for CLBP still needs further verification.

### Sensitivity analysis

**Fig. 8:**
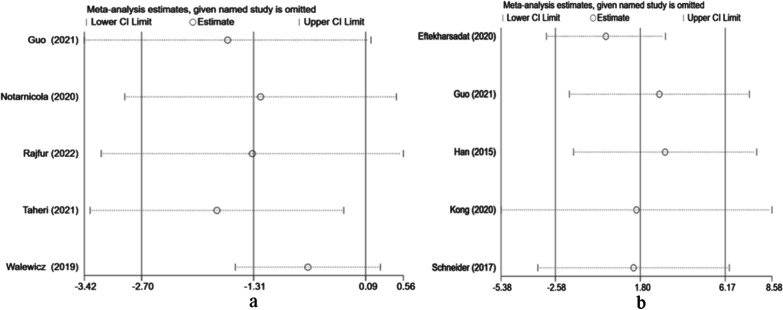
**a** Results of sensitivity analysis for VAS after omitting each study one at a time. CI: confidence interval. **b** Results of sensitivity analysis for the mental health score after omitting each study one at a time. CI: confidence interval

When comparing the effects of ESWT on pain at 12 weeks and mental health at 4 weeks, we performed a sensitivity analysis due to considerable heterogeneity. A single study was excluded each time to assess the impact of individual data on the overall outcome. The results showed that the merger effect was robust, and no significant deviation from the overall results was found in our study (Fig. 8).

### Publication bias

**Fig. 9:**
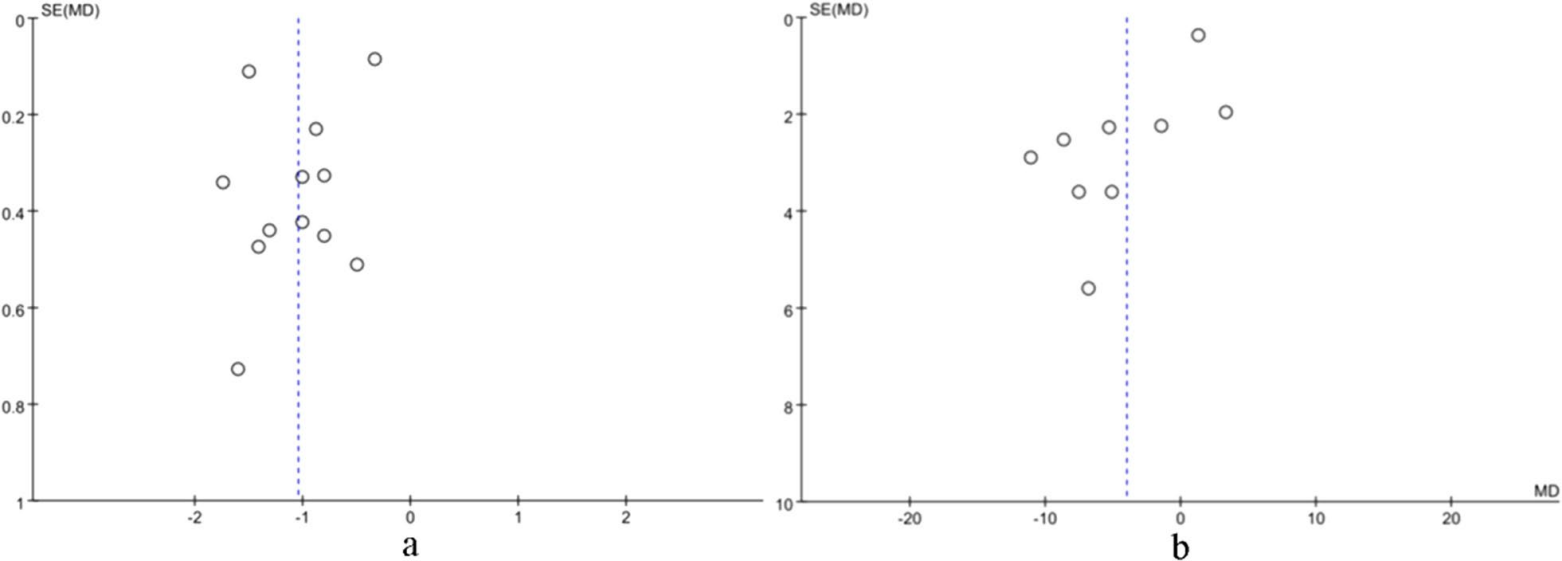
Funnel plot for the comparison of ESWT vs. Control at week 4. (**a**) Left figure: pain outcome as measured on a VAS. (**b**) Right figure: ODI outcome

Stata 15.0 software was used to conduct funnel plot analysis of the included literature on ESWT for 4-week pain and ODI outcome indices. The funnel plot showed a basically symmetrical scatter point, indicating that there was no significant publication bias in the included literature, and the results of the meta-analysis were credible (Fig. 9).

## Discussion

The effectiveness of ESWT on the pain, function and mental health of patients with CLBP was systematically reviewed. The results of this meta-analysis showed that ESWT, either as stand-alone or adjuvant treatment for CLBP, significantly reduced VAS scores at week 4 and week 12 compared to the control group, with a “moderate” recommended level based on GRADE [23]. Furthermore, “low” quality evidence showed significant improvement in ODI scores at week 4 and week 12 for ESWT compared to other conservative treatments. However, with regard to mental health scores at week 4, we did not find significant differences between the two groups. In addition, no ESWT-related adverse events were found (not recorded or did not occur) in any of the 12 RCTs included in the study.

According to our information, there was only one previous meta-analysis about the application of ESWT in CLBP, but we found that this study had high heterogeneity in both pain and dysfunction index analyses, and no subgroup analysis or sensitivity analysis was conducted [40]. This previous study found a nonsignificant effect of ESWT on pain relief in CLBP at 3 months, but our meta-analysis still found better long-term efficacy at 12 weeks after the inclusion of more studies. In addition, we found that this previous meta-analysis included an unpublished master's thesis and a study of participants with postpartum low back pain. These studies may have affected the reliability of the results, excluding them from our study. Finally, we included 12 RCTs with a total of 632 patients and explored the sources of the associated heterogeneity. Moreover, studies have shown that the occurrence of CLBP is rarely caused by a single factor but by a variety of physical and psychological mechanisms [41]. Holmes et al. [42] believed that limitations or disabilities in patients' daily lives would lead to psychosocial problems, which would further damage their quality of life. Therefore, we conducted the first meta-analysis of mental health scores in CLBP patients.

CLBP is treated with a variety of clinical approaches, including conservative treatment and surgical treatment. In its initial clinical use, ESWT was used by German medical scientists to save patients from surgical pain treatment [43]. With the passage of time, ESWT technology has gradually matured, and its clinical application is also increasing. Many clinical trials have shown that ESWT treatment can significantly reduce pain and complications in patients with CLBP [25, 32]. ESWT mainly treats chronic low back pain through the direct mechanical action of shock waves and indirectly causes mechanical action through cavitation [44]. First, when shock waves enter the human body, different mechanical effects will be generated at the interface of different tissues due to different contact media, such as fat, tendon, ligament and bone tissue, and finally, different forces will be generated on cells [45]. In these forces, tensile stress can relax tissues. It promotes microcirculation, while compressive stress can change the elasticity of cells and increase their ability to absorb oxygen for therapeutic purposes [46, 47]. Second, ESWT causes a large number of tiny bubbles to be created in the tissue, which rapidly expand and burst under the action of the shock wave, producing a high-speed fluid microjet and a shock effect [48]. This cavitation effect is particularly effective in reopening occluded microvasculature and releasing soft tissue adhesions at the joint [49, 50]. The exact mechanism of the pain-relieving and functional properties of ESWT is not fully understood, and several studies have attempted to elucidate the mechanisms of shock waves from basic science and clinical studies. Studies have shown that the energy released by ESWT is able to stimulate pain receptors located in skin, muscle, connective tissue, bone and joints and activate unmyelinated C and A delta fibres to initiate the "gated" pain control system and block nerve transmission, resulting in analgesic effects [51, 52]. In addition, ESWT has been shown to significantly downregulate the levels of IL-1, TNF-α and MMPs in degenerated joint tissues, thereby reducing the local inflammatory response [53, 54]. Additionally, ESWT also promotes the secretion of pain-reducing chemicals (e.g. endorphins), inhibits the release of pain factors such as substance P and calcitonin gene-related peptides, reduces peripheral nerve sensitivity and increases pain threshold levels [55, 56].

It is well known that adverse reactions are a major concern when evaluating the efficacy of ESWT. Therefore, the higher the risk of adverse reactions, the lower the clinical value of ESWT. In our study, no serious adverse reactions were reported in any of the 12 studies. Therefore, based on the current meta-analysis, ESWT did not increase the risk of local reactions. However, considering the small sample size included in the study, the safety of ESWT needs to be further discussed.

## Limitations

Some limitations of this study should be noted. First, there are differences in aetiology, pain duration, and related parameters used by ESWT in each study, which may lead to heterogeneity in the combination of results and limited evidence. Second, there are inevitably heterogeneous factors among the included patients, such as age, gender, and racial differences. Next, different biases, including selection bias, language bias, data provision bias and publication bias, may reduce the accuracy of the results. Last but not least, the pain, function and mental health scores included in this meta-analysis were all obtained through questionnaires, and the outcome indicators may be subjective. If there are enough articles with objective observation indicators in the future, relevant studies can be improved. Therefore, more RCTs need to be included in the future to further investigate the efficacy and safety of ESWT.

## Conclusion

ESWT is effective in reducing pain and dysfunction in CLBP patients without increasing the risk of adverse reactions, but it should be performed with caution. However, no significant effect was found on the improvement in mental health. More RCTs are needed to verify the findings in the current study.

## Supplementary Information

**Additional file 1**: Quality of evidence assessment by GRADE of the included studies.

## Data Availability

The datasets used and analysed during the current study are available from the corresponding author on reasonable request.

## Abbreviations

ESWT: Extracorporeal shock wave therapy
RCT: Randomized controlled trials
CLBP: Chronic low back pain
VAS: Visual analogue scale
ODI: Oswestry Disability Index
WMD: Weighted mean difference
SMD: Standardized mean difference
SD: Standard deviation
