# Supplementary material for: Efficacy and safety of extracorporeal shockwave therapy in chronic low back pain: a systematic review and meta-analysis of 632 patients
Source: J Orthop Surg Res. 2023 Jun 24;18:455. doi: 10.1186/s13018-023-03943-x (PMC10290808; doi:10.1186/s13018-023-03943-x)
Supplement: Supplementary file 1 — Additional file 1: Quality of evidence assessment by GRADE of the included studies. [file 13018_2023_3943_MOESM1_ESM.docx]

**Additional file 1**

**Table 2. Quality of evidence assessment by GRADE of the included studies.**

|  | | **GRADE Quality assessment** | | | | | |  |
| --- | --- | --- | --- | --- | --- | --- | --- | --- |
| Number of studies | Design | Risk of bias | Inconsistency | Indirectness | Imprecision | Publication Bias | Other considerations | Quality |
| **Pain at 4 weeks** (VAS) | | | | | | | | |
| 12 | RCTs | Serious^1^ | None Serious | None Serious | None Serious | Undetected | None Serious | ⊕⊕⊕◯  MODERATE |
| **Pain at 12 weeks** (VAS) | | | | | | | | |
| 5 | RCTs | Serious^2^ | None Serious | None Serious | None Serious | Undetected | None Serious | ⊕⊕⊕◯  MODERATE |
| **Disability at 4 weeks** (ODI) | | | | | | | | |
| 10 | RCTs | Serious^3^ | Serious^4^ | None Serious | None Serious | Undetected | None Serious | ⊕⊕◯◯  LOW |
| **Disability at 12 weeks** (ODI) | | | | | | | | |
| 4 | RCTs | Serious^5^ | None Serious | None Serious | Serious^6^ | Undetected | None Serious | ⊕⊕◯◯  LOW |

###### *Footnotes*

^1^ 12 studies were included. 6 study did not have a clear description of drop-outs (attrition bias). 1 study did not have a clear description of concealment of allocation (selection bias). 5 studies did not have clear description of patient blinding, 10 studies had problems with blinding of provider (performance bias) and 3 studies did not have a clear description of blinding of outcome assessor (measurement bias). Serious risk of bias.

^2^ 5 studies were included. 2 study did not have a clear description of drop-outs (attrition bias). 1 study did not have a clear description of concealment of allocation (selection bias). 1 study did not have clear description of patient blinding, 4 studies had problems with blinding of provider (performance bias) and 2 studies did not have a clear description of blinding of outcome assessor (measurement bias). Serious risk of bias.

^3^ 10 studies were included. 5 studies did not have a clear description of drop-outs (attrition bias). 1 study did not have a clear description of concealment of allocation (selection bias). 3 studies did not have clear description of patient blinding, 9 studies had problems with blinding of provider (performance bias) and 3 studies did not have a clear description of blinding of outcome assessor (measurement bias). Serious risk of bias.

^4^ Heterogeneity: *I*² = 96%, considerable heterogeneity; *Tau*² =22.02 (P < 0.01)

^5^ 4 studies were included. 1 study did not have a clear description of drop-outs (attrition bias). 1 study did not have a clear description of concealment of allocation (selection bias). 1 study did not have clear description of patient blinding, 4 studies had problems with blinding of provider (performance bias) and 2 studies did not have a clear description of blinding of outcome assessor (measurement bias). Serious risk of bias.

^6^ Total number of participants is insufficient (n = 139). The 95% CI did not include the no effect line.
